# Ichthyol in Respiratory Affections

**Published:** 1912-01

**Authors:** 


ICHTHYOL IN RESPIRATORY AFFECTIONS.—Ichthyol is a valuable drug in the treatment of affections of the respiratory tract. After the subsidence of an acute bronchitis it will act most advantageously. It may be given in peppermint water. Five minims is an average dose.—*Critic and Guide.*

